# Polarisation tuneable piezo-catalytic activity of Nb-doped PZT with low Curie temperature for efficient CO_2_ reduction and H_2_ generation†

**DOI:** 10.1039/d1na00013f

**Published:** 2021-02-15

**Authors:** Yan Zhang, Pham Thi Thuy Phuong, Nguyen Phuc Hoang Duy, Eleanor Roake, Hamideh Khanbareh, Margaret Hopkins, Xuefan Zhou, Dou Zhang, Kechao Zhou, Chris Bowen

**Affiliations:** State Key Laboratory of Powder Metallurgy, Central South University Hunan 410083 China; Institute of Chemical Technology, Vietnam Academy of Science and Technology TL29 Street, Thanh Loc Ward, District 12 HCM City Vietnam pttphuong@ict.vast.vn; Graduate University of Science and Technology, Vietnam Academy of Science and Technology 18 Hoang Quoc Viet Street, Cau Giay District Hanoi Vietnam; Department of Mechanical Engineering, University of Bath Bath BA2 7AY UK c.r.bowen@bath.ac.uk

## Abstract

The reduction of CO_2_ into useful hydrocarbon chemicals has attracted significant attention in light of the depletion in fossil resources and the global demand for sustainable sources of energy. In this paper, we demonstrate piezo-catalytic electrochemical reduction of CO_2_ by exploiting low Curie temperature, *T*_c_ ∼ 38 °C, Nb-doped lead zirconate titanate (PZTN) piezoelectric particulates. The large change in spontaneous polarisation of PZTN due to the acoustic pressures from to the application of ultrasound in the vicinity of the *T*_c_ creates free charges for CO_2_ reduction. The effect of applied acoustic power, particulate agglomeration and the impact of *T*_c_ on piezo-catalytic performance are explored. By optimization of the piezo-catalytic effect a promising piezo-catalytic CO_2_ reduction rate of 789 μmol g^−1^ h^−1^ is achieved, which is much larger than the those obtained from pyro-catalytic effects. This efficient and polarisation tunable piezo-catalytic route has potential to promote the development of CO_2_ reduction *via* the utilization of vibrational energy for environmental improvement.

## Abstract 2

First report on a detailed assessment of the impact of applied acoustic power, particle agglomeration effects and impact of *T*_c_ on piezo-catalysis for CO_2_ reduction.

## Graphical abstract

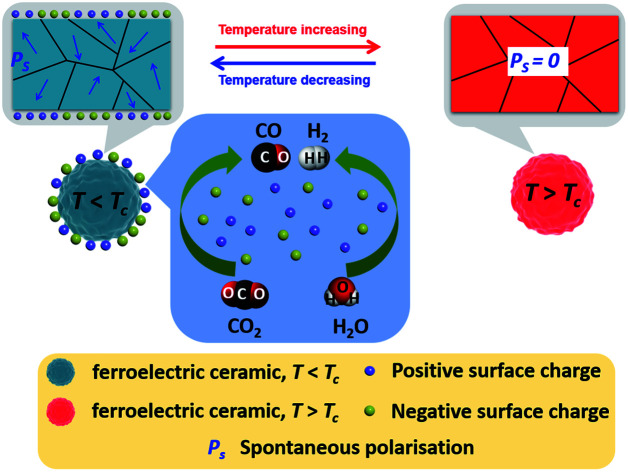

## Introduction

The increasing level of carbon dioxide (CO_2_) in the Earth's atmosphere is one of the greatest challenges facing humanity. Reducing the levels of atmospheric CO_2_ and minimising further release of CO_2_ into the atmosphere is key to tackling global warming and ocean acidification. Currently, the two main proposed solutions are (i) carbon capture and storage or (ii) carbon utilisation, the conversion of CO_2_ into useful chemicals. In the first case, CO_2_ is captured and sequestered for long-term storage, usually either geologic storage^1–3^ or ocean storage.^4,5^ Carbon utilisation, however, involves the conversion of CO_2_ into useful hydrocarbons, which has the potential to provide a sustainable and environmentally friendly solution that can tackle both the environmental problems and offer a sustainable source of energy for the high global demand. There are several routes to CO_2_ utilisation, including electrochemical CO_2_ reduction,^6^ hydrogenation^7^ and biological conversion.^8^ The process of CO_2_ reduction is a promising option for carbon capture and utilization, and can lead to the chemical conversion of CO_2_ into chemicals or fuels, such as formic acid (HCOOH), carbon monoxide (CO), ethylene (C_2_H_4_), ethanol (C_2_H_5_OH) and methane (CH_4_).^6–8^ While chemical reduction approaches are energetically costly due to the high stability of CO_2_, which necessitates high temperatures or high pressures, the electrochemical reduction of CO_2_ is gaining increasing interest and involves the conversion of CO_2_ to a more reduced chemical species using electrical energy. The products of the reaction are dependent on the catalyst used and operating reduction potential/voltage of the reaction.^6,7^ Electrochemical CO_2_ reduction is attractive for a number of reasons. Firstly, the process can operate at ambient pressure and temperature. Secondly, there is potential to use renewable energy sources, such as solar or wind, to provide the electrical energy. Thirdly, the controllability, modularity and scale-up of the methods can be relatively simple.^6–8^ Therefore, electrochemical CO_2_ reduction has the potential to provide a sustainable, long-term solution to help manage and reduce atmospheric CO_2_ levels.

In this paper we explore the use of a ferroelectric material to achieve piezo-catalytic CO_2_ reduction. Ferroelectric materials have a diverse range of applications, including sensors, actuators, resonators and, more recently, piezo-catalysis.^9–11^ Recent work by Kakekhani *et al.* has focused on the use of ferroelectric ceramic materials for pyro- and piezo-catalysis.^12–14^ The change in polarisation of ferroelectric materials with stress can create charge to induce piezo-catalysis, which has potential in energy harvesting and environmentally sustainable technologies.^15^ Piezo-catalysis operates by converting mechanical energy into chemical energy where polarisation induced charges and free carriers are used to catalyse reduction–oxidation (red–ox) reactions. Therefore, it has potential to be an electrode-free and cost-effective process compared to existing electro-catalysis techniques. In addition, Kakekhani *et al.* stated that the changing polarisation level of a ferroelectric can influence the adsorption and desorption processes due to its different surface states,^14^ with potential to overcome some of the limitations of catalytic efficiency due to the Sabatier principle.^16^ A detailed overview of the surface electro-chemistry of piezo-catalysis has been provided by Starr *et al.*^17–19^ Piezo-catalysis have several advantages compared to existing photo-catalysis processes; they can operate in dark conditions and can harvest low-frequency vibrations present in the environment^20^ to drive electrochemical reactions.

Piezo-catalysis has attracted interest for wastewater treatment, dye degradation,^21–24^ water splitting for hydrogen (H_2_) generation^22,25–27^ and piezoelectric–photocatalytic dye degradation.^28–33^ Piezo-catalytic water splitting for H_2_ generation is of interest as a sustainable alternative to current routes, such as steam methane reforming, which can be energy intensive, reliant on fossil fuels and produce CO_2_ as a by-product.^34^ While the use of ferroelectric semiconductor catalysts for photocatalytic CO_2_ reduction^35^ and pyro-electro-catalytic CO_2_ reduction^36^ has been demonstrated, there is limited work on demonstrating piezo-catalytic CO_2_ reduction, which is explored in this paper.

In terms of materials for piezo-catalysis, a range of materials have been investigated. Lead zirconate titanate [Pb(Zr_*x*_Ti_1−*x*_)O_3_, PZT] ceramics with the perovskite structure (ABO_3_) are widely employed as piezoelectric materials for sensors, actuators, and energy related applications due to their remarkable ferroelectric and piezoelectric properties.^37^ The piezoelectric properties can be regulated by compositional modifications using dopants or modifiers. Donor doping, such as the addition of Nb^5+^ on the B-site (Ti^4+^ and/or Zr^4+^), leads to the production of lead vacancies to maintain charge neutrality, which can ‘soften’ the ferroelectric properties of the PZT system and reduce the Curie temperature (*T*_c_) or coercive and enhance the dielectric properties.^38,39^ While the effects of Nb-doping in PZT on properties have been reported,^40–44^ the investigation on the application of the Nb-doped PZT on piezo-catalysis performance is in its infancy.

In this paper we will demonstrate the potential of using Nb-doped PZT as a low Curie temperature ferroelectric material for piezo-catalytic CO_2_ reduction in the presence of ultrasound. Of particular interest is the potential to use Nb-doping to reduce the Curie temperature (*T*_c_) below 100 °C since it allows the level of piezo-catalysis to be investigated above the *T*_c_ (where there is no spontaneous polarisation) and approaching the *T*_c_ (where there is a spontaneous polarisation) to examine its impact on the progression of electrochemical reduction. The use of piezo-catalysis in the vicinity of the *T*_c_ is considered an novel approach to enhance piezo-catalytic activity.^45^ Careful experiments are undertaken to separate the piezo-catalytic contribution to the overall reaction from any sono-chemical contributions that originate from ultrasonically induced cavitation events that create localised heat for chemical energy conversion. This has enabled a detailed assessment of the impact of applied acoustic power, particle agglomeration effects and impact of *T*_c_ on piezo-catalysis for CO_2_ reduction; which has yet to be reported. The potential for piezo-catalysis for H_2_ generation is also discussed since the two reactions occur simultaneously within the reactor system.

## Method

### Material and chemicals

Pb_0.99_(Zr_0.95_Ti_0.05_)_0.98_Nb_0.02_O_3_ (PZTN) powders were prepared by a solid-state reaction. Analytical grade (Sigma Aldrich) lead oxide (Pb_3_O_4_, 99%), zirconium dioxide (ZrO_2_, 99%), niobium oxide (Nb_2_O_5_, 99.9%) and titanium dioxide (TiO_2_, 99.9%) were selected as starting materials and weighed according to their stoichiometric ratio with a 0.5 wt% excess of Pb_3_O_4_ to compensate for volatilization during sintering. The above mixtures were calcined at 1000 °C for 3 h, followed by additional ball-milling for 12 h. The milled powders were mixed with 1 wt% poly(vinyl alcohol) (PVA) binder and dried in an oven at 60 °C. To manufacture dense materials for ferroelectric characterisation, the powders were uni-axially cold-compacted to form pellets of 10 mm in diameter and 1 mm in thickness. The pellets were first heated to 600 °C for 3 h to remove the binder and then sintered at 1200 °C for 2 h. Na_2_SO_3_ and α-Al_2_O_3_ were purchased from Merck. Double distilled water was employed to prepare the sample solutions. 20 mL precision screw-thread headspace vials with round bottom (22.5 × 75.5 mm) were purchased from Asia Laboratory Instruments Co., Ltd. (Vietnam).

### Materials characterisation

The chemical states of the synthesized powders were analyzed by X-ray photoelectron spectroscopy (XPS, ESCALAB250Xi, Thermo). The binding energies were calibrated utilizing the C 1s peak (284.8 eV) as an internal standard. Scanning Electron Microscopy (SEM) was performed using a FEI Quanta 250 FEG Quanta scanning electron microscope. X-ray diffraction (XRD) analysis was carried out on the Rigaku D-Max/2550VB+ (Cu Kα radiation *λ* = 1.5418 Å) X-ray diffractometer. Piezo Force Microscopy (PFM) using the contact mode of an atomic force microscope (NanoManTM VS) was undertaken with a conductive Pt/Ir-coated Si cantilever (SCM-PIT) to investigate the piezo-response phase and amplitude of the dense ferroelectric sample.

### Experimental setup and procedure for piezo-catalysis

**Fig. 1:**
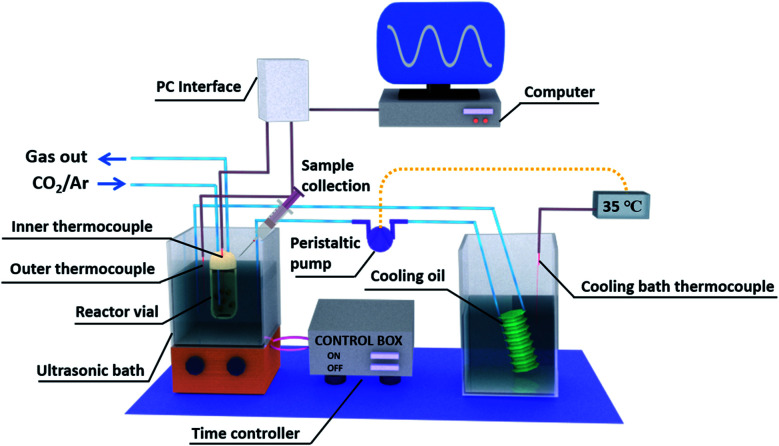
Schematic of the complete experimental setup for piezo-catalysis based on a double-bath-type sonoreactor.

Fig. 1 shows a schematic image of the complete experimental setup for piezo-catalysis assessment, which is based on double-bath-type sonoreactor. Sonolysis was conducted in an ultrasonic cleaner bath (Taiwan Total Meter) operating at 40 kHz. Typically, 10 mL of 0.1 M Na_2_SO_3_ solution acting as hydroxyl radical (OH*) scavenger was poured into a 20 mL headspace vial prior to being added with a known amount of catalyst and fitted with a septum cap. Before being sonicated, the solution was sequentially saturated with pure argon and 2% CO_2_ diluted in argon for at least 40 min. A specific cover was used to keep the test vial in a desired position to study the effect of vial position on the efficiency of sonolysis, see Fig. S1.† The temperature of the ultrasonic bath (*T*_bath_) was controlled at a desired temperature (±0.5 °C) by circulating water through an external cooling bath during the reaction time (*t*_react_). Since the experiments were conducted under a pulse mode where both the ultrasound pulse length and pulse interval were fixed at 15 s, the actual sonication time (*t*_US_) is half of the reaction time. The operating temperature of the reactor was controlled by changing the water bath temperature, which led to a change in the average working temperature. As a result of the applied acoustic energy, the average working temperature is higher than the water bath temperature. The working temperature of the reactor was varied from temperatures approaching the Curie temperature (*T*_c_), where the material is ferroelectric, to temperatures above *T*_c_, where the material is paraelectric and no longer ferroelectric/piezoelectric. Clearly, we would expect a decrease in piezo-catalytic activity above the *T*_c_.

The amount of generated gases was measured after 30 min of reaction by a gas chromatograph (HP 5890 Series II, Agilent), which was equipped with a thermal conductivity detector (TCD) and used argon as a carrier gas. An RT-Msieve 13X capillary column with dimension of 30 m in length and 0.32 mm internal diameter (Thames Restek) was used to separate H_2_, O_2_, N_2_, CH_4_ and CO. No other carbon-based products that may be formed from CO_2_ reduction were detected using a gas chromatograph (HP 5890 Series II, Agilent) equipped with a thermal conductivity detector (TCD), a flame ionization detector (FID) and a HP-PLOT U capillary column with dimension of 30 m in length and 0.53 mm internal diameter (Agilent J&W) used helium as a carrier gas. It was found that CO was the only product of CO_2_ reduction, thus, quantification of the gaseous fuels was conducted using ChemStation Software based on the calibration curves which were previously determined by using different concentrations of hydrogen and CO diluted in argon. The uncertainties of gaseous fuel productions have been determined by calculating their standard deviations from at least two experiments.

Experiments were conducted using a range of piezo-catalyst dosage levels, from 0 (as a zero dose control), 0.1, 0.3, 0.5, 0.7 and 1 g L^−1^. In order to provide an additional control, a non-ferroelectric power (Al_2_O_3_) was studied as the same dosage levels since the presence of particles in the solution can also influence the acoustic power.

The addition of particles, and their agglomeration, can have a significant impact on the acoustic power within the reactor and is therefore carefully calibrated. The sono-chemical contribution is quite different to piezo-catalysis, involving kinetic/vibrational energy from cavitation events during the application of ultrasound to create heat for chemical energy conversion. In this regard, the agglomeration of particles at high power dosage levels can reduce the acoustic power delivered to the reactor. The acoustic power (*P*_A_, which is expressed in W) absorbed by a known amount of water in the reactor vial and ultrasonic bath was estimated by calorimetric measurements^46^ using the experimental setup shown in Fig. S1(B),† and calculated as follows:1
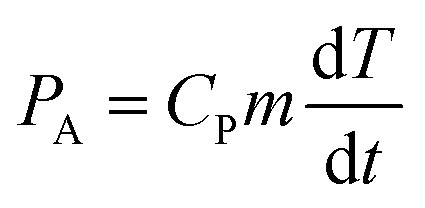
where *C*_P_, *m* and d*T*/d*t* are the heat capacity of water at constant pressure (4.178 J g^−1^ K^−1^), the mass of water (g), and the ramping rate of temperature (K s^−1^), respectively. Fig. S2† shows the temperature profiles of calorimetric measurements for the determination of acoustic intensity in the reactor vial that was placed at different locations in a vertical direction (*z* = 10, 13, 17, and 27 mm, as indicated in Fig. S1†). Accordingly, the thermal ramp rate was determined from the slope of the temperature–time line during the first 20 s.^46^ It can be seen that the highest acoustic power was obtained at *z* = 17 mm, thus this height was selected for all further experiments in this paper. Consequently, the acoustic intensity (*I*_A_, which is expressed in W L^−1^) was calculated as the acoustic power divided by the volume of the irradiated liquid. All calorimetric measurements were performed in triplicate.

**Fig. 2:**
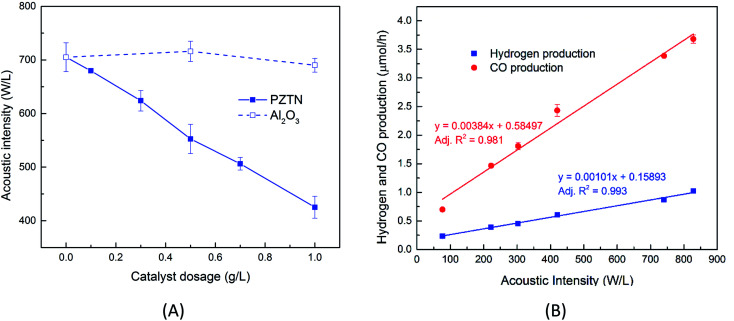
(A) Effect of catalyst particle dosage on the delivered acoustic intensity into the reactor; (B) relationship between acoustic intensity and sono-chemical production of hydrogen and carbon monoxide (*T*_bath_ = 25 °C, *t*_react_ = 30 min). In this case the sample contains zero dosage of particles.

By performing calorimetry measurements at different catalyst dosages, it was found that increasing the PTZN dosage resulted in a significant decrease in acoustic intensity, whereas only a small linear change was observed in the case of the non-ferroelectric alpha-Al_2_O_3_ control; this can be seen in Fig. 2A. The agglomeration of PTZN particles (see Fig. S3†) act to prevent the ultrasonic wave being absorbed inside the reactor, resulting in a decrease in acoustic intensity. The greater agglomeration of PTZN, as compared to the non-ferroelectric Al_2_O_3_, may result from the different particle morphology, surface chemistry or the generation of piezoelectric charges under the application of ultrasound.

In addition, there is a strong relationship between the acoustic intensity inside the reactor and the sono-chemical activity both in terms of hydrogen production and CO_2_ reduction. This can be observed from the zero dosage experiments (no particulates added), as can be seen in Fig. 2B. Increasing the 40 kHz acoustic intensity leads to an increase in free radical production, which is mainly due to an increase in bubble collapse temperature, bubble collapse time, and amount of water vapour trapped in the bubble.^47–49^ It has been reported that there is an upper limit of intensity, above which no sono-chemical activity can be observed.^50,51^ A comparative study that was performed using a 20 kHz and 1 MHz sonoreactor showed that different cavitation conditions resulted in different effects of acoustic intensity, thus, this threshold should be experimentally determined for each case.^51^ In our study, Fig. 2B shows both CO and H_2_ production rates exhibit a strong linear relationship with acoustic intensity, which agrees well with other studies,^52–54^ and indicates that the critical intensity of this 40 kHz sonoreactor is higher than 830 W L^−1^. Both CO and H_2_ data are reported since there is likely to be a competition between CO_2_ reduction and hydrogen production from piezo-catalytic water splitting where water molecules are oxidised by the charge to form H^+^ and O_2_, which can generate H_2_ or reduce CO_2_ to CO and H_2_O; this will be quantified later in the paper.

The reaction products in the sonoreactor will be formed from a combination of sono-chemical and piezo-catalytic contributions and the data in Fig. 2A and B can be used to determine each contribution to the overall reaction products. From Fig. 2A, the higher the dosage of PZTN, the lower the delivered acoustic intensity into the vial reactor. As a result, there will be a smaller contribution from sono-chemical CO and H_2_ production, using the data in Fig. 2B. The piezo-catalytic contribution to production rate at different catalyst dosages can be estimated as follows:

2 *r*^piezo-catalytic^_i_ = *r*^sono-piezo^_i_ − *r*^sono-chemical^_i_

where *r*^piezo-catalytic^_i_ is the piezo-catalytic production rate at a specific acoustic intensity, *r*^sono-piezo^_i_ is the measured production rate of component i which is a combination of piezo-catalytic and sono-chemical contributions and *r*^sono-chemical^_i_ is the sono-chemical production rate of the component i, which can be estimated from the correlation equations from Fig. 2B:3
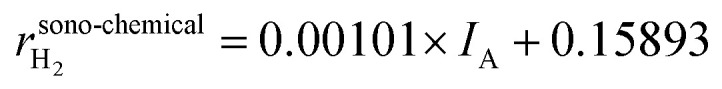

4 *r*^sono-chemical^_CO_ = 0.00384 × *I*_A_ + 0.58497

where *I*_A_ is the average acoustic intensity which was delivered into the reaction vial in the presence of PZTN at the corresponding catalyst dosage and obtained from Fig. 2A.

We now report the experimental results, which will provide an initial characterisation of the Nb-doped lead zirconate titanate materials to determine its ferroelectric properties and Curie temperature (*T*_c_) since the aim is to conduct piezo-catalysis below and above the *T*_c_. Hydrogen generation and CO_2_ reduction are then described, with particular emphasis on the impact of dosage level of the reaction products, determining the contribution of sono-chemical and piezo-catalytic activity. The impact of reactor temperature in relation of *T*_c_ will be assessed. Finally, a mechanism for the process in these discussed.

## Results and discussion

**Fig. 3:**
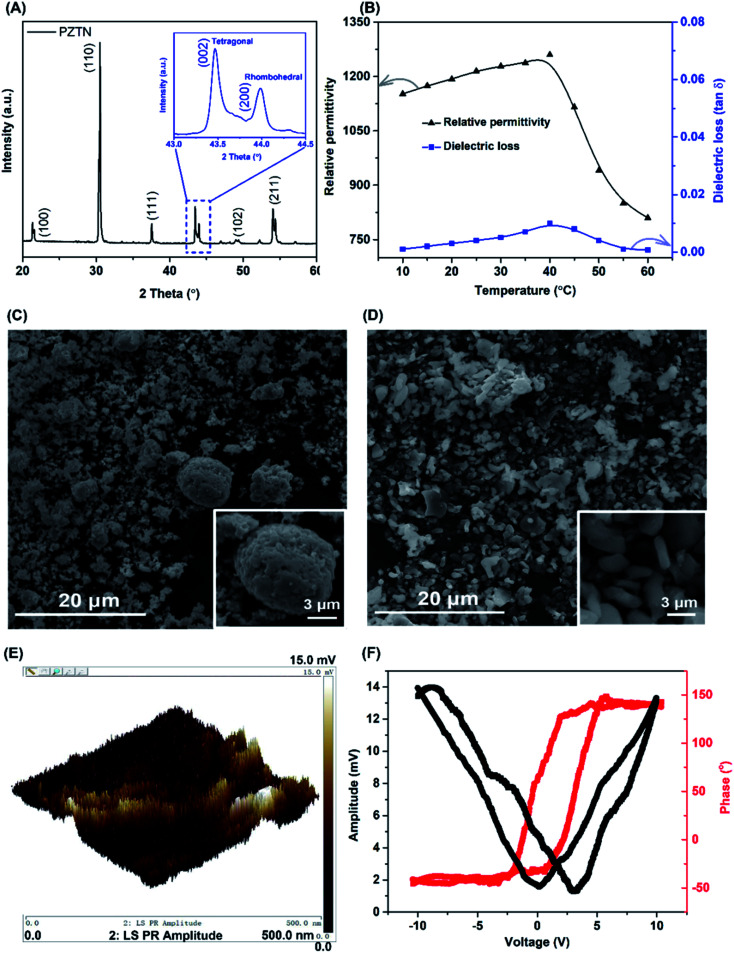
(A) XRD pattern of Nb-doped PZT powders (PZTN) powders. (B) Relative permittivity as a function of the temperature. SEM images of (C) PZTN and (D) Al_2_O_3_ (control) powders. PFM spectrum on the local evidence of the existence of ferroelectricity in the Nb-doped PZT powders (E) out-of-plane PFM amplitude and (F) local hysteresis loop behaviour for the amplitude and phase.

Fig. 3A shows the room temperature X-ray diffraction patterns of the PZTN powders with the two theta (2*θ*) ranging from 20° to 60°. The sharp and well-defined single-phase diffraction peaks confirm the presence of a perovskite crystal structure, with negligible peaks from impurity phase. The insets of Fig. 3A show evidence of peak splitting at 2*θ* near ∼43.5°, indicating the coexistence of non-centrosymmetric tetragonal (002) and rhombohedral (200) phases in perovskite structure, which can be attributed to the presence of Nb^5+^ as the B-site donor dopant.^55–57^Fig. 3B shows the temperature dependence (10 to 60 °C) of the relative permittivity (*ε*_r_) and dielectric loss (tan *δ*) for the PZTN dense ceramics at 1 kHz. As expected, the relative permittivity and the dielectric loss reach a maximum at the transition at the Curie temperature (*T*_c_ ∼ 38 °C) from the ferro-paraelectric phase to the paraelectric phase, with the values of *ε*_r_ ∼ 1259 and tan *δ* ∼ 0.009, respectively. The permittivity and loss then decreased with a further increase in temperature, as a result of symmetry change from the ferroelectric tetragonal/rhombohedral phase to the symmetric and paraelectric cubic phase. Fig. 3C and D present SEM micrographs of the PZTN and alpha-Al_2_O_3_ (used as a control) powders after the application of continuous ultrasound for 1 hour in the ethanol. It can be seen that PZTN powders consisted mainly of large spherical particles with a diameter of ∼9 μm (see the inset of Fig. 3C) and some smaller-sized spherical-like particles (∼2 μm), while the Al_2_O_3_ powders exhibit a smaller size variation, but a more irregular morphology with a length and width of ∼3 μm and ∼0.6 μm, respectively. Fig. 3E and (F) show the local ferroelectric domain switching in the PZTN powders from piezo force microscopy. The dark brown and light-yellow contrast in Fig. 3E and S4(A)† represent the signatures of the potential different polarization vectors of ferroelectric domains. A ‘butterfly’ loop in the amplitude–voltage response as the saturation voltage reached ± 8.4 V was observed, indicating the PZTN powders has ferroelectric and piezoelectric properties. A phase angle–voltage hysteresis of the PZTN powders is also shown in Fig. 3F and S4(B).† The phase angle exhibits a ∼189° change under the reversal of the dc bias electric field, confirming a polarization domain switching process and is another indicator of ferroelectricity from the PZTN powder. In addition, the macro-scale polarisation–electric field (*P*–*E*) hysteresis loop of an unpoled dense PZTN disk can be observed in Fig. S5,† with of coercive field of 11 kV cm^−1^ and a spontaneous polarisation of ∼30 μm cm^−2^, but is less square than the work of Chen *et al.*,^58^ since an unpoled sample was measured in our work.

**Fig. 4:**
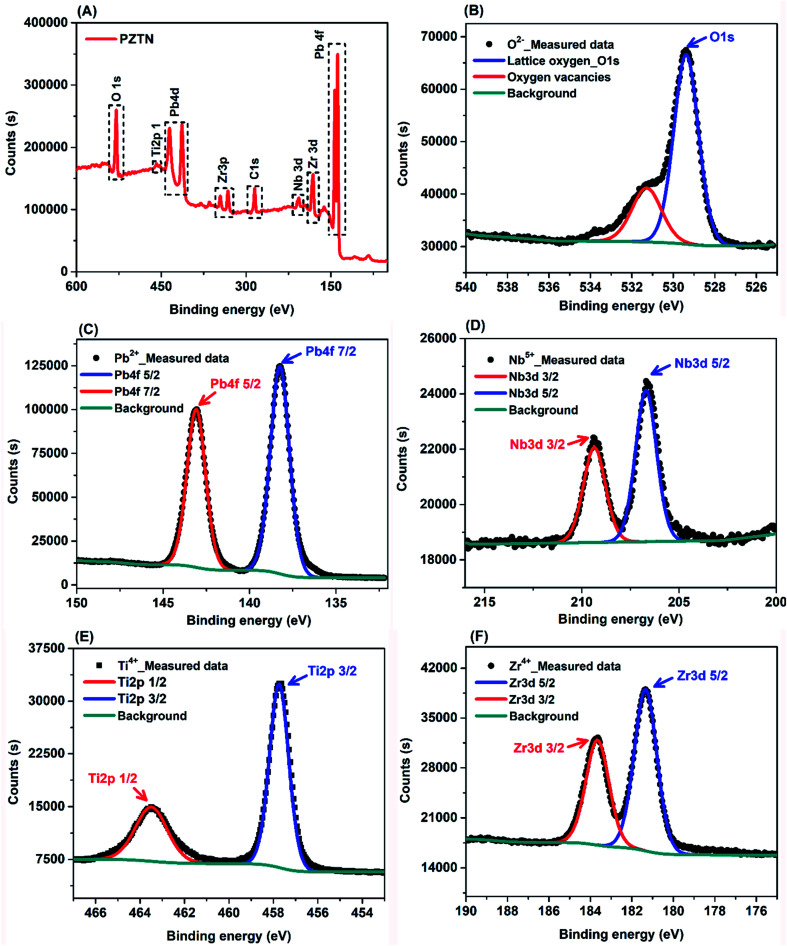
(A) XPS survey spectrum obtained from the PZTN powders, and high-resolution spectrum of (B) O 1s, (C) Pb 4f, (D) Nb 3d, (E) Ti 2p, (F) Zr 3d.

Fig. 4A shows a full scan XPS spectrum of the synthesized PZTN powders to analyse their surface composition and chemical state. The broad spectrum further corroborate that they are multicomponent metal oxides composed of Pb, Zr, Ti, Nb and O, which is in good agreement with the XRD result in Fig. 3. The Pb 4f_7/2_, Zr 3d_5/2_ Ti 2p_3/2_, and Nb 3d_5/2_ peaks represent their fully oxidized states, namely Pb ion in its Pb^2+^, Zr ion in its Zr^4+^, Ti ion in its Ti^4+^ and Nb ion in its Nb^5+^ state.^59,60^ In addition, there are also peaks from C, which are likely to be due to powder being exposed to air and molecules being adsorbed from the atmosphere, such as carbon dioxide, that contribute to the C peak intensity. Fig. 4B shows O 1s signal and corresponding curve fitting. The higher peak with the binding energy of ∼529.00 eV corresponds to oxygen in the PNZT lattice, including Pb–O, Ti–O, Zr–O, and Nb–O bonding, whereas the binding energy of 531.43 eV (the lower red curve) originates from oxygen vacancies on the surface as a result of hydroxylation due to the samples being exposed to an ambient atmosphere.^61,62^Fig. 4C illustrates XPS spectra of the Pb 4f signal and the corresponding curve fitting. The fitted Pb 4f peaks showed predominantly a contribution from oxidized Pb (Pb^2+^), and the deconvoluted double peaks of Pb 4f_7/2_ and Pb 4f_5/2_ were located at the binding energies of 138.19 eV and 143.09 eV, respectively. The Pb^2+^ peak that is shifted to a higher binding energy is consistent with the Nb-doped PZT film.^63^ The spin–orbit split Pb (4f) peak exhibits only one binding state at Pb (4f_7/2_) ∼ 138.31 eV and Pb (4f_5/2_) 143.18 eV, which corresponds to the state of lead in the perovskite lattice,^64,65^ demonstrating that only one chemical state has been realized in the synthesized powders. As shown in Fig. 4D, the convoluted HR-XPS spectrum of Nb 3d is composed of doublets corresponding to spin orbital splitting of 3d_5/2_ and 3d_3/2_ observed at 207.0 eV and 209.9 eV, revealing the existence of Nb^5+^. In the spectrum of Ti 2p, shown in Fig. 4E, the Ti 2p_3/2_ and Ti 2p_1/2_ peaks are observed at binding energies of 457.89 and 463.49 eV, indicating the chemical state of Ti^4+^. The high resolution Zr 3d spectrum (Fig. 3F) can be deconvoluted to two sub-peaks, where two main peaks at 183.68 and 181.39 eV are ascribed to the Zr 3d_3/2_ and Zr 3d_5/2_ transitions, respectively. This indicates that Zr ions in the powders are in the Zr^4+^ chemical state. Since the surface chemistry of a catalyst determines its catalytic activity,^66–68^ their chemical composition and states can dramatically affect their catalytic properties. For the piezo-catalytic chemical reactions, the surface of the ferroelectric PZTN material is the platform of the piezo-catalytic reaction, where the Pb, Zr, Ti, Nb, and O ions found by XPS and its ferroelectric nature can provide active sites for piezo-catalysis under the application of the ultrasound, with some carbon or hydroxylation from the atmosphere or water.

### Effect of catalyst dosage of hydrogen and CO production

**Fig. 5:**
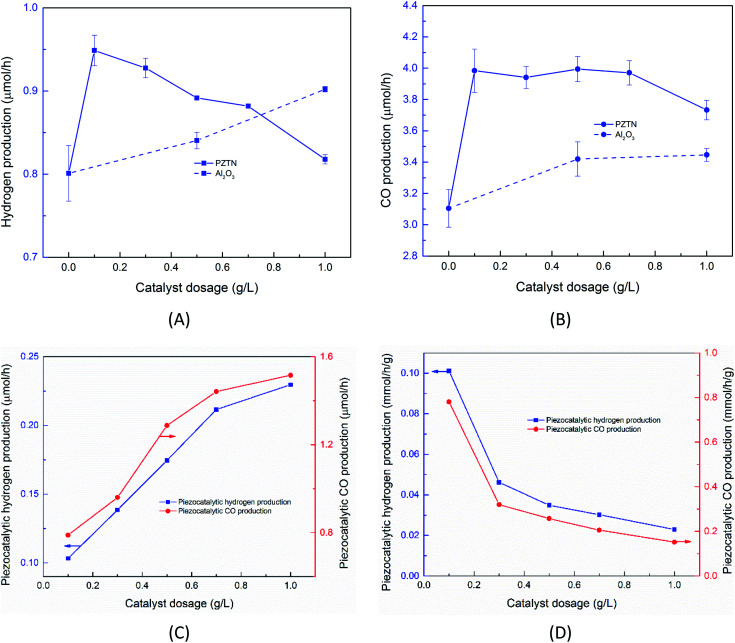
(A) Effect of catalyst dosage on the sono-piezo catalytic hydrogen and (B) CO production rate (*T*_bath_ = 25 °C, *t*_react_ = 30 min); (C) effect of PZTN catalyst dosage on the piezo-catalytic production of hydrogen and carbon monoxide expressed in units of μmol h^−1^, and (D) mmol h^−1^ g^−1^.

Fig. 5A shows the effect of catalyst dosage on the hydrogen and CO production rate with a water bath temperature at room temperature (*T*_bath_ = 25 °C) for a reaction time of *t*_react_ = 30 min. It should be highlighted that the data in Fig. 5A is likely to contain reaction products formed from a combination of sono-chemical and piezo-catalytic contributions. The ferroelectric PTZN is the piezo-catalytic material and the non-ferroelectric Al_2_O_3_ is the sono-chemical control; the zero powder dosage level is an additional sono-chemical control. For the alumina control a small increase in CO and H_2_ can be observed for the case of using non-ferroelectric alpha-Al_2_O_3_ particles; this is possibly due to the presence of non-ferroelectric particles in the solution enhancing the nucleation of cavitation events and resulting sono-chemical activity. Meanwhile, while the particle size of PTZN is similar to that of non-ferroelectric alpha-Al_2_O_3_, it behaves differently under the application of ultrasound. It can be seen that the addition of a small amount of PTZN (∼0.1g L^−1^) can significantly increase the generation of both hydrogen and carbon monoxide compared to the zero powder dosage level. However, increasing the concentration beyond 0.1 g L^−1^ of PTZN did not further enhance the CO and H_2_ production. The reason for the low increase in CO and H_2_ of PTZN at higher catalyst dosage levels is likely to be the agglomeration of ferroelectric PZTN particles, see Fig. 2A and S5,† which can limit the generation of charges at the surfaces under sonication. This hypothesis is in good agreement with the reduction in acoustic density with PZTN dosage in Fig. 2A. Using the data in Fig. 2A and equation from Fig. 2B, it is possible to subtract the sono-chemical contribution to hydrogen and CO production in Fig. 5A and B at different catalyst dosages to determine the piezo-catalytic contribution. This piezo-catalytic data is presented in Fig. 5C and D. The data in Fig. 5C indicates that the total piezo-catalytic products (μmol h^−1^) for PTZN increases with an increase in catalyst dosage. However, Fig. 5D shows that the production rate per gram of PTZN (mmol h^−1^ g^−1^) decreases with an increase in the amount of catalyst as a result of increased agglomeration, which leads to a reduction in acoustic intensity, as was observed in Fig. 2B.

**Fig. 6:**
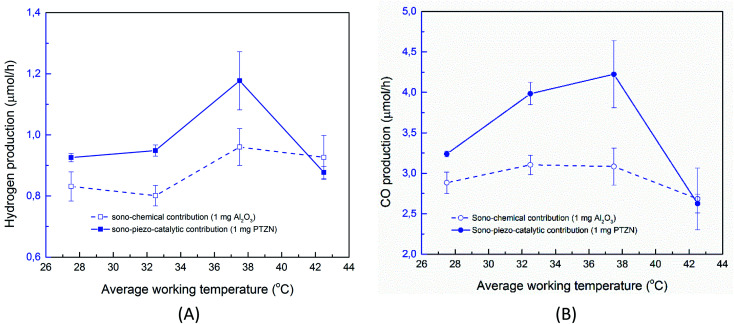
(A) Effect of working temperature on the hydrogen and (B) CO production rate (catalyst dosage 0.1 g L^−1^, *t*_react_ = 30 min).

While previous work has shown the efficiency of a sonoreactor is affected by the bulk liquid temperature,^69,70^ there are limited studies investigating experimentally its effect on the sono-chemical activity both in terms of CO and H_2_ production. As stated, there is likely to be a competition between CO_2_ reduction and hydrogen production formed *via* water splitting. In addition, the working temperature should strongly affect the piezo-catalytic contribution, since above the *T*_c_ ∼ 39 °C the material is in a non-ferroelectric cubic phase (Fig. 3B) and the piezo-catalytic contribution should be small. The operating temperature was varied by changing the water bath temperature, which led to a change in the average working temperature that is typically higher than the water bath temperature due to the additional acoustic energy. Fig. 6A and B shows the production rate of CO and H_2_ at different working temperatures. It can be seen that the sono-chemical hydrogen and CO production rate (in the presence of 1 mg non-ferroelectric Al_2_O_3_) were only slightly affected by temperature whereas its influence on the piezo-catalytic rate (in the presence of 1 mg ferroelectric PZTN) was greater in the vicinity of *T*_c_. It has been theoretically demonstrated that increasing the bulk liquid temperature simultaneously results in a decrease in the bubble collapse temperature and an increase in vapour pressure of volatile components.^69,70^ While the latter effect can enhance the formation of free radicals from the dissociation of the water vapour molecules, the former one may cause a decrease in their decomposition into free radicals. Consequently, both previous simulation and experimental works have revealed that there is an optimum working temperature at which maximum rates are obtained.^69–72^ These effects were experimentally found to be more pronounced in the presence of an organic compound compared to pure water^71–73^ which is in agreement with our results. Accordingly, the optimum water bath temperature at which the highest rate of sono-chemical hydrogen and CO productions were observed is 30 °C (see Fig. S4†), which is in agreement with 30–40 °C reported by other studies.^69,71–75^ In addition, the highest and most distinct sono-piezo-catalytic hydrogen and CO production were observed at a working temperature of 38 °C, which correlates with the *T*_c_ of PZTN, as shown in Fig. 3B. It has previously been discussed that pyro-catalytic H_2_ generation activity is greatest near the *T*_c_,^22^ and this work demonstrates that is also applied to CO production. By optimization of the piezo-catalytic effect of PZTN in relation to its *T*_c_ and in the presence of ultrasound, a promising piezo-catalytic CO_2_ reduction rate of 789 μmol g^−1^ h^−1^ is achieved, which is much larger than the those obtained from the pyro-catalytic effect using Bi_2_WO_6_ (16.5 μmol g^−1^ h^−1^).^35^

### Mechanism for piezo-catalysis

**Fig. 7:**
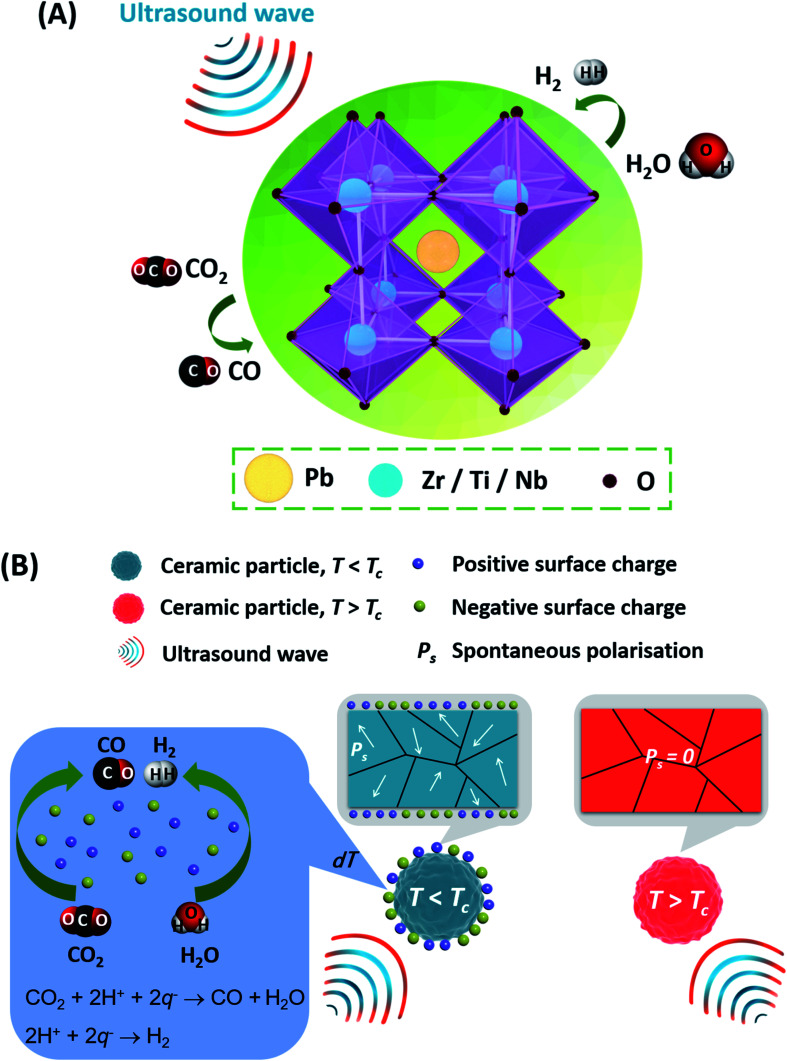
Schematic of mechanism for CO_2_ reduction and H_2_ generation based on the application of ultrasound vibrations to ferroelectric PZTN. (A) electrochemical reactions during ultrasonic excitation, (B) enhanced CO production and H_2_ generation achieved due to piezo-catalytic and sono-chemical effects when *T* < *T*_c_.

Fig. 7 is used to elucidate the possible CO_2_ reduction and H_2_ generation pathway offered by the ferroelectric PZTN powders. Electrochemical reactions can occur in the presence of ultrasonic waves, as shown in Fig. 7A, based on a combination of the piezoelectric and sono-chemical contributions. Specifically, charges can be excited from the surface of each ferroelectric particle based on its change in spontaneous polarisation (*P*_s_) when a periodic ultrasonic acoustic pressure (*σ*) is applied to initiate redox reactions, as shown in Fig. 7B where the powder particle can be single or multi-domain (as seen in the figure). The critical redox absolute potentials for CO_2_ reduction and water splitting are similar in terms of ∼0.38–0.61 V^76,77^ and ∼1.23 V,^78^ respectively. While it is difficult to measure the local surface potential of the single piezoelectric particle during the ultrasound, our recent work^22^ has demonstrated that the barium strontium titanate (Ba_0.75_Sr_0.25_TiO_3_) with the comparable ferroelectric polarisation has the ability to split the water for the H_2_ generation with the application of ultrasound. The piezoelectric potential can be estimate by the equation,^79^
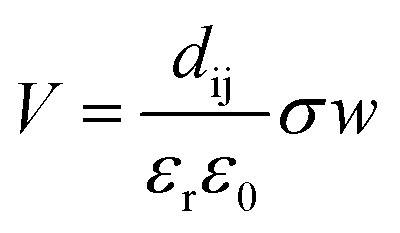
where *V* is the piezoelectric potential, *d*_ij_ is the piezoelectric charge coefficient, *ε*_r_ is relative permittivity, *ε*_0_ is permittivity of the free space, *σ* is the applied stress and *w* is the particle width. Based on the piezoelectric charge coefficient (*d*_33_ ∼ 69 pC N^−1^ ^80^ and relative permittivity (*ε*_r_ ∼ 305 ^80^ of PZTN, a particle size (*w* = 2 μm, see Fig. 3), and a cavitation stress of ∼10^8^ Pa,^81^ the piezoelectric voltage is *V* ∼ 51 V, well above the critical redox potential. While there is potentially a decrease in voltage on increasing temperature as a result of an increase in permittivity close to *T*_c_, the level of charge *Q* (*Q* ∝ *d*_ij_*σw*^2^) for reduction will reach a maximum at *T*_c_, since *d*_ij_ is a maximum close to *T*_c_.^82^ The different electronic properties and thermodynamic ground states of negatively-poled and paraelectric surfaces of ferroelectrics have also been shown to influence catalysis, with potential for depolarisation under stress near *T*_c_.^83^

When *T* < *T*_c_, surface charges are released which results in a piezoelectric potential and increased CO production and H_2_ generation by piezo-catalysis in combination with sono-chemical processes; see centre of Fig. 7B. When the temperature is increased to *T* > *T*_c_, CO and H_2_ production is reduced since it is a result of only sono-chemical effects where the energy barrier to form cavitation bubbles were reduced;^84^ corresponding to the right had side of Fig. 7B. The change in spontaneous polarisation of the ferroelectric PZTN powders facilitates the separation of electric charge carriers along the polarization direction and their migration toward the surface. The charges and ions involved in the CO_2_ reduction and H_2_ generation by reactive species such as *q*^−^, *q*^+^, and H^+^, are shown in eqn (5)–(8). Charges with an opposite sign (*q*^−^, *q*^+^) are generated during the application of ultrasound to ferroelectric PZTN (eqn (5)), where all the surface charges would be released when *T* is in the vicinity of *T*_c_. Water molecules are then oxidised by *q*^+^, to form H^+^ and O_2_ (eqn (6)), while together with the *q*^−^, it can generate H_2_ (eqn (7)) and reduce CO_2_ to CO and H_2_O (eqn (8)). As a result, there is a strong competition between CO_2_ reduction (eqn (8)) and hydrogen production (eqn (7)) from water splitting. The results showed that piezo-catalytic CO/H_2_ production ratio was greater than 7.6, exhibiting that the reaction 8 for CO production is dominant over the reaction (7) for H_2_ production. Further work can involve characterisation of the products in solution, for example by high performance liquid chromatography (HPLC) or high resolution nuclear magnetic resonance (HNMR) and examining the role of changing the concentration of Na_2_SO_3_ hydroxyl radical (OH*) scavenger.5
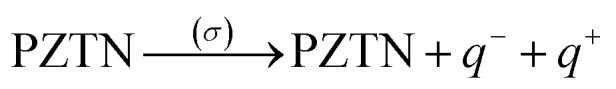

6 H_2_O + 2*q*^+^ → 2H^+^ + ½O_2_

7 2H^+^ + 2*q*^−^ → H_2_

8 CO_2_ + 2H^+^ + 2*q*^−^ → CO + H_2_O

## Conclusions

The paper provides the first demonstrated of the capability of low Curie temperature Nb-doped lead zirconate titanate powders for CO_2_ reduction using the piezo-catalytic effect, with quantification of its dominance over H_2_ generation. The change of the polarisation was the driving force to realise the chemical reactions, in terms of CO_2_ reduction and water splitting processes, when the ultrasound was applied. Careful experiments were undertaken to elucidate the impact of powder dosage level and agglomeration on the applied acoustic pressure. It is also shown that it is necessary to separate the sono-chemical and piezo-catalytic contributions to the overall reaction products. The critical acoustic intensity of the 40 kHz sonoreactor is higher than 830 W L^−1^ in this case and the piezo-catalytic contribution was greatly reduced when the working temperature higher than the Curie temperature of ∼39 °C, confirming the impact of the piezoelectric and ferroelectric nature of PZTN on catalysis. A catalyst dosage with an optimum addition of 0.1 g L^−1^ was achieved, where higher dosage levels can lead to agglomeration and a reduction in acoustic pressure that limits charge generation under sonication. A promising piezo-catalytic CO_2_ reduction rate of 789 μmol g^−1^ h^−1^ was achieved, which is larger than that obtained from pyro-catalysis of Bi_2_WO_6_ (16.5 μmol g^−1^ h^−1^).^35^ As the electromechanical coupling factor (*k*_p_) is a measure of the effectiveness with which a piezoelectric material converts mechanical energy into electrical energy or converts electrical energy into mechanical energy, the piezoelectric ceramics (*e.g.* PZT, BaTiO_3_, PZTN, *etc.*) with a *k*_p_ ranging from 0.15 to 0.6 exhibit good capability to contribute to the conversion of electrical energy to chemical energy. Future work will focus on and evaluation of the whole efficiency of the system, and the following approaches to improve efficiency can include: (i) improving the dispersion of the ferroelectric powder near the Curie temperature to achieve high particle dosage levels at high levels of acoustic pressure and improve the amount of products formation, (ii) evaluation of 3D porous piezoelectric material in order to reduce agglomeration of powders; (iii) further control of the *T*_c_*via* compositional control of the ferroelectric, and (iv) coating of the ferroelectric particles with transition metals to further facilitate catalysis.

## Conflicts of interest

There are no conflicts to declare.

## Supplementary Material

NA-003-D1NA00013F-s001
