# Supplementary material for: Polarisation tuneable piezo-catalytic activity of Nb-doped PZT with low Curie temperature for efficient CO2 reduction and H2 generation
Source: Nanoscale Adv. 2021 Feb 15;3(5):1362–74. doi: 10.1039/d1na00013f (PMC9418403; doi:10.1039/d1na00013f)
Supplement: NA-003-D1NA00013F-s001 [file NA-003-D1NA00013F-s001.pdf]

Electronic Supplementary Material (ESI) for Nanoscale Advances.
This journal is © The Royal Society of Chemistry 2021

Supplemental

Polarisation tunable piezo-catalytic activity of Nb-doped PZT with low Curie temperature for efficient H_2 generation and CO_2 reduction

Yan Zhang[1, *], Pham Thi Thuy Phuong[2, 3, *, #], Nguyen Phuc Hoang Duy[2], Eleanor Roake[4], Hamideh Khanbareh[4], Margaret Hopkins[4], Xuefan Zhou[1], Dou Zhang[1], Chris Bowen[4, #]

1 State Key Laboratory of Powder Metallurgy, Central South University, Hunan, 410083, China

2 Institute of Chemical Technology, Vietnam Academy of Science and Technology, TL29 Street, Thanh Loc Ward, District 12, HCM City, Vietnam

3 Graduate University of Science and Technology, Vietnam Academy of Science and Technology, 18 Hoang Quoc Viet Street, Cau Giay District, Hanoi, Vietnam

4 Department of Mechanical Engineering, University of Bath, Bath BA2 7AY, UK

* Those authors contributed equally.

[#] Corresponding authors: pttphuong@ict.vast.vn; c.r.bowen@bath.ac.uk

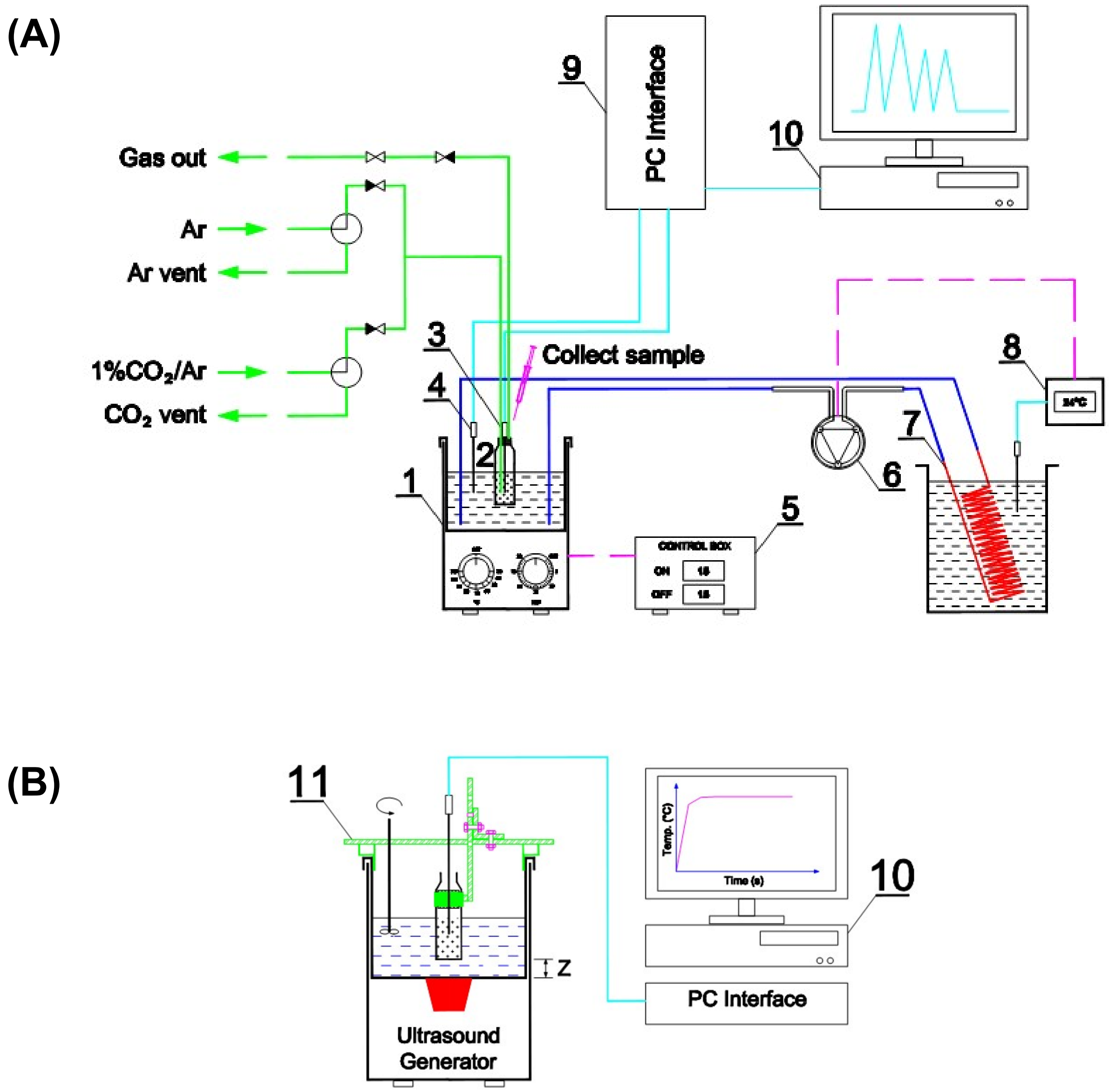

Figure S1. (A) Complete experimental setup for piezo-catalysis based on double-bath-type sonoreactor, and (B) experimental setup for calorimetric measurement.

1 - Ultrasonic bath; 2 – Reactor vial; 3 – Inside thermocouple; 4 – Outside thermocouple; 5 – Timer controller; 6 – Peristaltic pump; 7 – Cooling coil; 8 – Cooling bath thermocouple; 9 – Interface; 10 – Computer; 11 – Specific cover

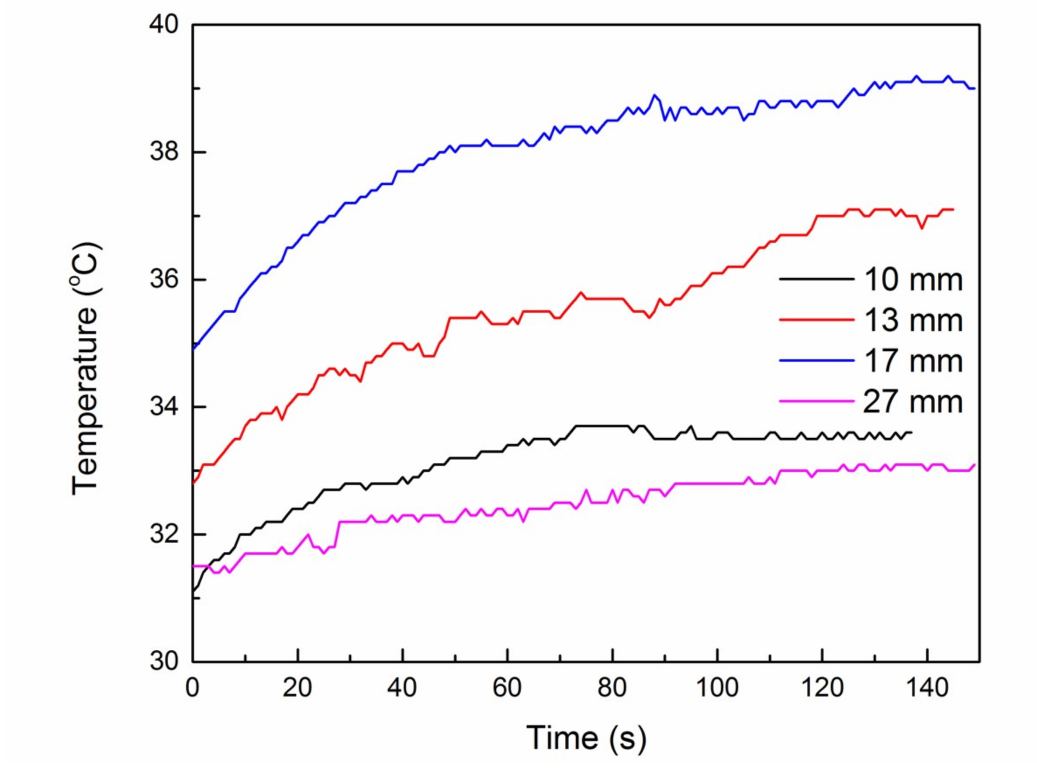

Figure S2. Temperature profiles of calorimetric measurements at different height locations of

z = 10, 13, 17, and 27 mm, as indicated in Figure S1(B).

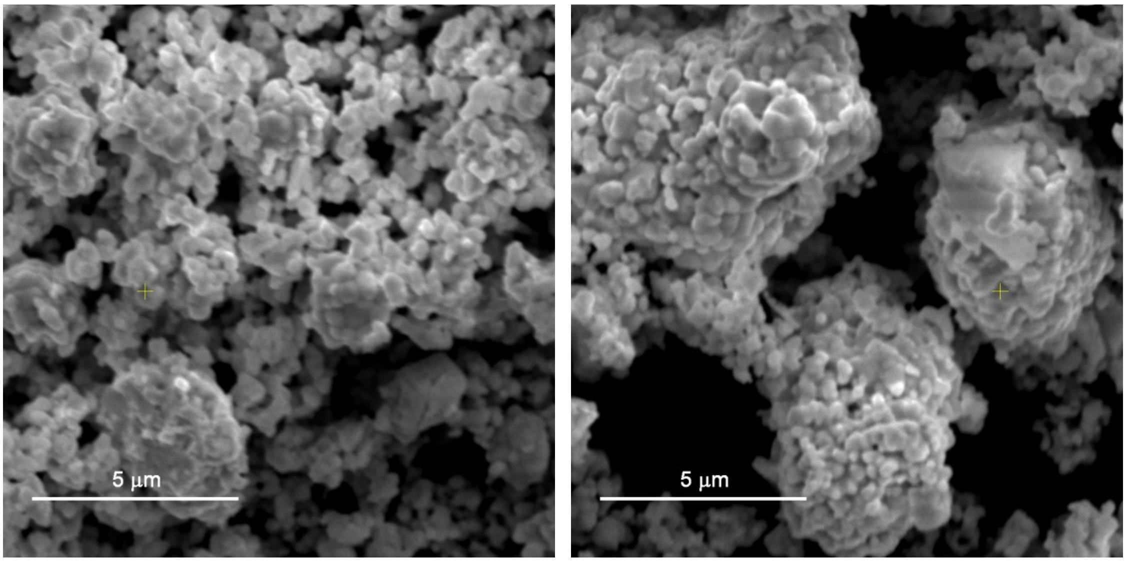

(A) (B)

Figure S3. SEM images of the used PZTN powders at dosage of (A) 0.1 g/L. and (B) 1.0 g/L

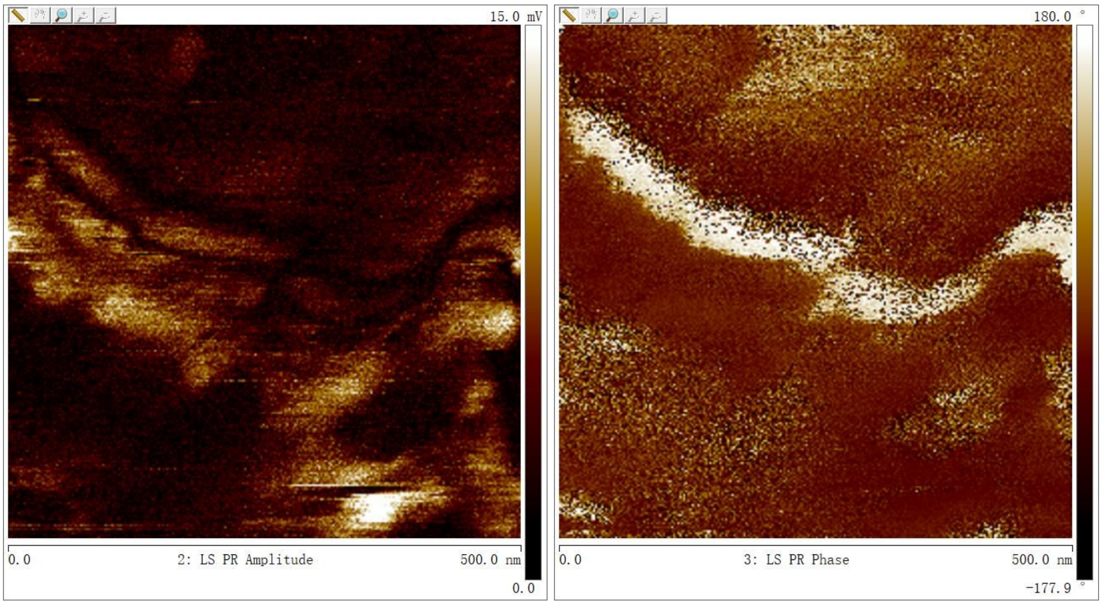

Figure S4. PFM images of PZTN powders. (A) 2D Amplitude image, (B) PFM phase image.

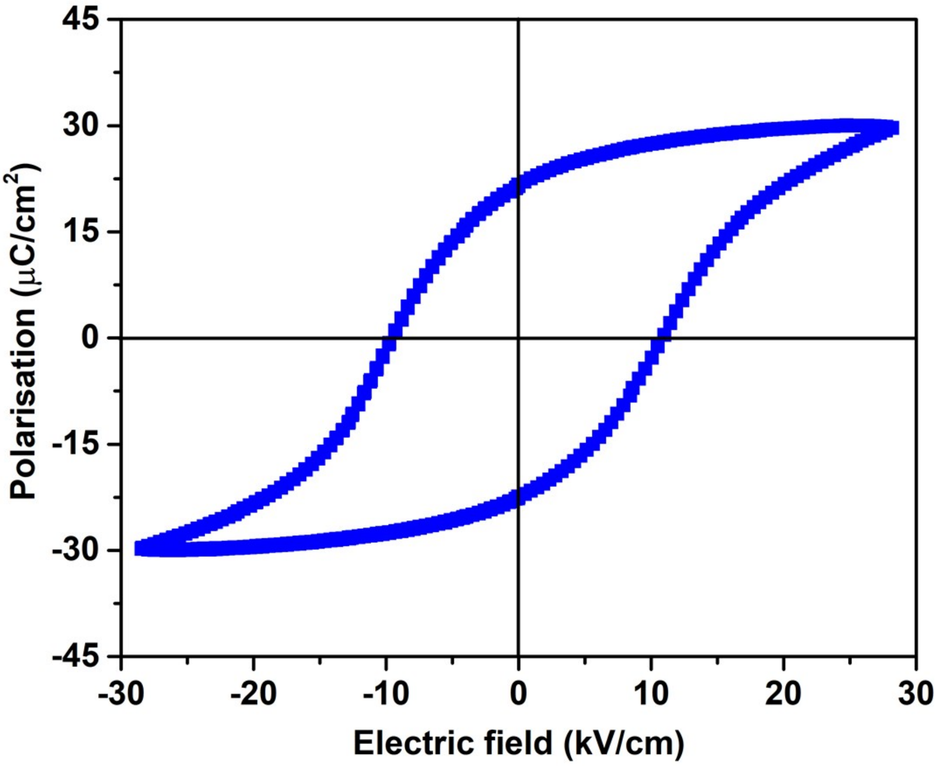

Figure S5. Room-temperature P-E hysteresis loop of the unpoled dense PZTN disk.

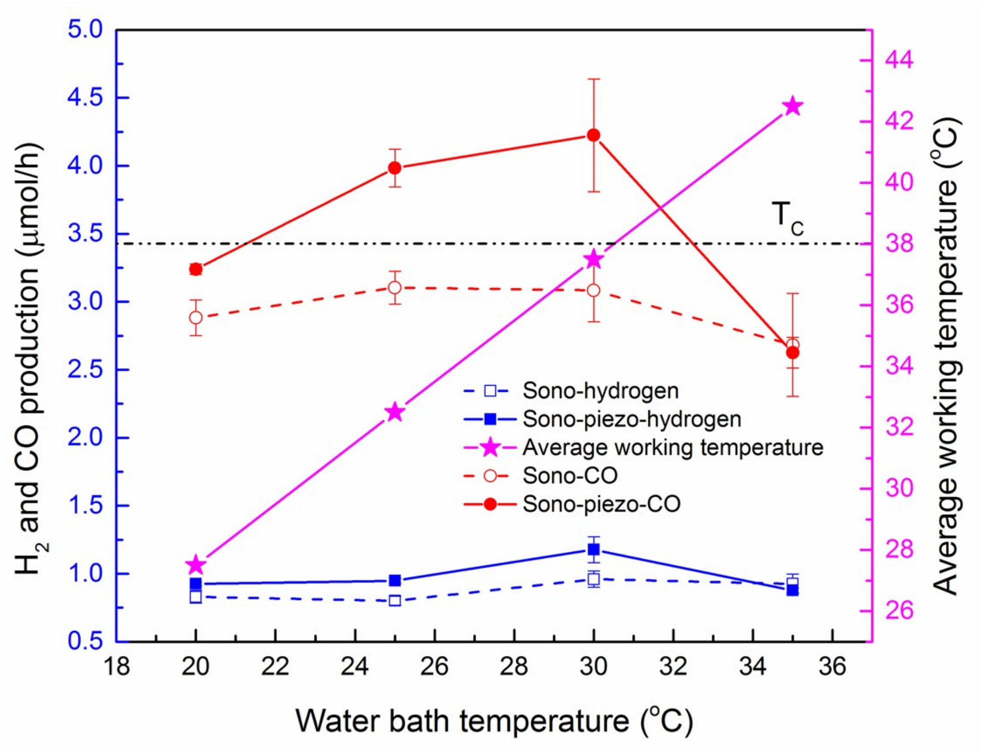

Figure S6. Effect of water bath temperature on the hydrogen and CO production rate (catalyst dosage 0.1g/L , t_{react} = 30 min)
